# Selective REM-Sleep Deprivation Does Not Diminish Emotional Memory Consolidation in Young Healthy Subjects

**DOI:** 10.1371/journal.pone.0089849

**Published:** 2014-02-27

**Authors:** Jarste Morgenthaler, Christian D. Wiesner, Karoline Hinze, Lena C. Abels, Alexander Prehn-Kristensen, Robert Göder

**Affiliations:** 1 Department of Psychiatry and Psychotherapy, Christian-Albrechts University School of Medicine, Kiel, Germany; 2 Department of Child and Adolescent Psychiatry and Psychotherapy, Christian-Albrechts University School of Medicine, Kiel, Germany; 3 Institute of Psychology, Christian-Albrechts University, Kiel, Germany; University of Texas at San Antonio, United States of America

## Abstract

Sleep enhances memory consolidation and it has been hypothesized that rapid eye movement (REM) sleep in particular facilitates the consolidation of emotional memory. The aim of this study was to investigate this hypothesis using selective REM-sleep deprivation. We used a recognition memory task in which participants were shown negative and neutral pictures. Participants (N = 29 healthy medical students) were separated into two groups (undisturbed sleep and selective REM-sleep deprived). Both groups also worked on the memory task in a wake condition. Recognition accuracy was significantly better for negative than for neutral stimuli and better after the sleep than the wake condition. There was, however, no difference in the recognition accuracy (neutral and emotional) between the groups. In summary, our data suggest that REM-sleep deprivation was successful and that the resulting reduction of REM-sleep had no influence on memory consolidation whatsoever.

## Introduction

Sleep and emotion modulate memory consolidation, and the role of sleep in memory consolidation is supported by a multitude of studies [1]–[2]. Similar effects of sleep on consolidation have been found in most memory systems, including emotional memory. For example, Wagner *et al.* [3] identified a selective benefit on retrieval of content of an emotional story when compared to retrieval of content of a neutral story, if sleep was in the retention interval. Payne *et al.* [4] showed their participants scenes with neutral or negative objects in front of a neutral background. Unlike a retention interval spent awake, a retention interval spent asleep led to selective consolidation of the negative objects at the cost of memory of the neutral background. In summary, there are several studies in support of the hypotheses that emotional aspects of memory relative to neutral aspects of memory are selectively enhanced by sleep [5]. On the other hand, Lewis *et al.* [6] found contradicting results. The authors showed their participants pictures with either negative or neutral contexts and compared the recognition performance after wake and sleep retention intervals. Their data showed a smaller decay of context memory in the course of sleep than during wakefulness; however emotional and neutral context memories were protected to the same extent during sleep. However, they were able to record a stronger increase in activity in brain areas specific for emotional processing, such as the left amygdala and right parahippocampus, during correct retrieval of negative contexts after sleep.

Recent papers discuss the function of different sleep phases in memory consolidation. Specifically, it is hypothesized that slow-wave sleep primarily enhances the consolidation of hippocampus-dependent, declarative memories [1]–[2]. In addition, REM sleep is believed to selectively enhance the consolidation of emotionally tagged memory content while attenuating the emotion itself [5]. In line with the earlier study by Wagner and colleagues [3], Groch *et al.* [7] showed better recognition of emotional stimuli than of neutral stimuli after late, REM-rich nocturnal sleep compared to early, slow-wave rich sleep (SWS). Furthermore, correlations between REM-sleep during a short nap [8] or overnight sleep [9] and the consolidation of emotional memory have been reported. In contrast, some recent studies failed to replicate any selectively enhancing effect of REM-sleep on the consolidation of emotional memory. Hu *et al.* [10] also did not find a benefit for emotional relative to neutral stimuli when they asked for “remember” judgments; the assumed influence of REM-sleep was only reproducible for “know” judgments. In a recognition task of negative and neutral stimuli, Baran *et al.* [11] could not identify any relationship between emotional memory and measures of REM-sleep; they were, however, able to identify such a relationship for emotional salience. In summary, there is some evidence in favor of the hypothesis that REM sleep fosters the consolidation of emotional memory. It is unclear, however, whether REM sleep is necessary for the consolidation of emotional memory.

To the best of our knowledge, this is the first study to investigate the effect of selective REM-sleep deprivation on emotional memory consolidation. Emotional memory was tested using negative pictures of the IAPS [12]. Since most of the recent studies using other study-designs suggested a link between REM sleep and emotional memory performance (3, 7–10), we hypothesized that selective REM-sleep deprivation would result in decreased emotional memory retention.

## Materials and Methods

### Participants

All participants gave written informed consent before participating. The study was approved by the local ethics committee of the School of Medicine of the Christian-Albrechts University of Kiel (proposal no. A 449/11). Each participant was compensated with payment for participating in this study. Participants were 39 medical students recruited at the University of Kiel campus. Exclusion criteria were a history of neurologic or psychiatric disorders, sleep disorders, medications, or a body-mass-index above 30. Another exclusion criterion was a recognition performance of less than .7 in the encoding control of the memory paradigm. Neurologic or psychiatric symptoms were measured by self–report and the German version of the standardized symptom checklist (SCL-90-R) [13]. Two of the main scores of the SCL-90-R, the Global Severity Index (GSI), and the Positive Symptom Total Score (PST) were used. The GSI is an indicator for general psychological stress, while the PST reflects the number of symptoms. For both scores, participants with results outside the average were excluded (T-values: <40 or >60). Sleep disturbances were screened with the Pittsburgh Sleep Quality Index (PSQI) [14]. A cut-off-score ≥5 suggested by Buysse et al. [14] was used to exclude participants with unhealthy sleep. Because of a possible influence on the sleep architecture [15], samples were limited to right-handed participants, as measured by self-report and the Edinburgh Handedness Inventory [16], and non-smokers [17]. The Digit Span Forward test from the WMS-R [18] was used to make sure that concentration and working memory of all participants were average at minimum. The Digit Span test took place before the learning phase of the memory paradigm.

The data of 6 participants could not be used for further analysis, either due to technical problems during data recording or because the participants were not able to fall asleep. The data of 2 more participants were discarded because they had too much REM-sleep within the experimental group (40.5 min) or insufficient REM- sleep within the control group (8.53 min), respectively. 2 more participants failed the encoding control test and were therefore excluded (for details, see below). The final sample as the basis of the data analysis consisted of 29 participants (18 female, 11 male) aged between 19 and 25 (mean 23, SD = 1.71). 14 participants were in the control group and 15 participants were REM-sleep deprived.

The participants' age in both groups, handedness, and self-reported sleep quality did not differ significantly between groups. There were also no significant differences between the experimental groups in the GSI and PST scores from the SCL-90-R (Table 1).

**Table 1:** Group comparisons between REM-deprivation group and control group.

	REM-deprivation group Mean (SEM)	Undisturbed sleep group Mean (SEM)	*t*	*df*	*p*
Age	22.73(0.396)	23.29 (0.507)	0.87	27	.395
PSQI	3.00 (0.258)	3.21 (0.261)	0.58	27	.560
GSI	39.07 (2.333)	40.29 (2.543)	0.35	27	.726
PST	41.33 (2.233)	41.71 (2.694)	0.11	25.73+	.914
EDHI	0.98 (0.050)	0.89 (0.26)	−1.26	13.94+	.229

PSQI, Pittsburgh Sleep Quality Index; GSI, Global Severity Index (part of the SCL-90-R); PST, Positive Symptoms Total score (part of the SCL-90-R); EDHI, Edinburgh Handedness Inventory;

+ significant Levene-Test for equal variances.

### Procedure

The participants spent two nights in our sleep laboratory. The first night's purpose was to exclude severe sleep disorders like sleep apnea syndrome and to adapt the participants to the conditions in the sleep laboratory. At 10:00 p.m. before the second night in the laboratory, the participants were shown the stimulus pictures (encoding phase) and then immediately tested with a small subsample of pictures afterwards to ensure sufficient encoding (encoding control test). Sleep was recorded between lights off (approx. 10:40 p.m.) and lights on in the morning (approx. 6:45 a.m.). The participants performed the recognition test at 8:00 a.m.. Participants were randomly assigned to either the control or the experimental group (gender was parallelized). Participants in the experimental group were REM-deprived during the experimental night, while the sleep of participants in the control group was undisturbed. One week before or after the sleep condition (order counterbalanced), a wake condition was employed. For this purpose, the participants arrived at 9:00 a.m. (+/−1 h) and attended a parallel version of the learning phase and encoding control test of the memory paradigm. They were asked to spend the following retention interval awake (even without napping). At 07:00 p.m. (+/−1 h) the recognition test phase of the memory paradigm followed.

### Memory Task

Stimuli were two sets of 260 pictures (130 emotional and 130 neutral). Most of them were taken from the International Affective Picture System (IAPS) [12], whereas a few pictures (20%) were chosen from an in-house picture set which included images similar to the IAPS set. These pictures were used in the studies of Prehn-Kristensen et al. [19], [20] and approved in this research. The emotional pictures from the IAPS ranged in their arousal from 4.46 to 7.26 on a 9-point-scale (from 1 not arousing, to 9 maximal arousing) and in their valence from 1.48 to 4.85 (9-point-scale from 1 negative, to 9 positive). Means and standard deviations for the emotional pictures were 5.72±0.72 (arousal) and 2.81±0.69 (valence) and for the neutral pictures 3.33±0.78 (arousal) and 5.47±0.76 (valence). Picture selection was orientated to the IAPS-norms [12]. Both picture sets were parallelized with respect to arousal and valence ratings. The pictures in both sets were presented in pseudorandom order and the order of presentation was same for all participants, who were aware that this was a memory test. During the *learning phase* 130 pictures (65 emotional and 65 neutral) were presented on a computer screen for 1.5 s each. Participants were asked to rate the arousal of each picture on a 9-point scale (SAM) [12], [21]. Afterwards, the participants completed the *encoding control test*. The encoding control stimuli included 20 pictures (10 emotional and 10 neutral) from the learning phase (targets) mixed with 20 novel pictures (10 emotional and 10 neutral) (distractors). Every stimulus from the encoding control was shown for 1.5 s followed by an old/new memory judgment. The encoding control did not include enough pictures for a reliable analysis of a recognition baseline. Therefore the results of the encoding control were only used to assure that the participants complied with the instructions, attended to the task, and showed a sufficient encoding performance (accuracy rate cut off .7). Exploratory t-tests revealed no significant differences in encoding performance between experimental groups and between conditions. The retention interval was followed by the final *recognition test* in which 220 pictures were shown for 1.5 s each, and the participants were asked for an “old”/”new” judgment without a time limit for their answer. The 110 targets from the learning phase (55 emotional and 55 neutral) were mixed with 110 previously unseen distracters (55 emotional and 55 neutral). Accuracy (difference between hit-rate and false alarm-rate) was used as a measure of memory performance [7]. In addition we calculated the sensitivity d′ and the response bias c (see Supplements S1).

Participants of both experimental groups were asked to rate their individual emotional state once after sleep before the memory retrieval on a 9-point valence (1 =  happy, pleased, content, optimistic; 9 =  unhappy, bugged, discontent, sad, desperate) and arousal (1 =  relaxed, calm, lazy, sleepy; 9 =  excited, frantic, nervous, wide awake, aroused) scale (SAM) [21]. We introduced these measures to control for possible effects of sleep inertia on memory retrieval. The consideration of these covariates in the ANOVA did not change the outcome and that is why they are not reported on below. There were no significant differences concerning emotional state in the morning between participants of both experimental conditions as shown in Table 2.

**Table 2:** Group comparisons between REM-deprivation group and control group for short-term memory and the emotional state after the experimental night.

		REM-deprivation group (Mean(SEM))	Undisturbed sleep group (Mean (SEM))	t	df	p
**Digit span before encoding**	wake condition	11.40 (0.505)	10.86 (0.376)	−0.85	27	.402
	sleep condition	11.13 (0.424)	10.07 (0.412)	−1.792	27	.084
**Participants emotional state after sleep**	valence rating	5.13 (0.559)	6.57 (0.510)	1.89	27	.069
	arousal rating	2.93 (0.419)	3.36 (0.308)	0.805	27	.428

Short-term memory is measured by the digit span total score; emotional state of the participants is measured by the ratings on the 9-point self-assessment manikin (SAM) valence (1 =  happy, pleased, content, optimistic; 9 =  unhappy, bugged, discontent, sad, desperate) and arousal (1 =  relaxed, calm, lazy, sleepy; 9 =  excited, frantic, nervous, wide awake, aroused) scales.

### Sleep Recording and Deprivation

Sleep during the experimental night was recorded by standard procedures using a digital electroencephalogram (EEG), electromyogram (EMG), electrooculogram (EOG), and electrocardiogram (EKG). The EEG montage according to the 10–20 system included the positions C4 referenced to A1, O2 referenced to A1, and F4 referenced to A1. F3 referenced to A2, C3 referenced to A2, and O1 referenced to A2 were used as backup positions. To record sleep parameters, the polysomnographic recoding-system SOMNOscreen PSG plus TM (SOMNOmedics, Randersacker, Germany) was used. Data were analyzed according to the specifications provided in the revised AASM manual [22] by a certified rater unaware of the hypotheses. Sleep spindles (11–16 Hz) were visually identified in all epochs scored as N2-sleep. Spindles exceeded 0.5 seconds and had a typical waxing and waning spindle morphology. Sleep spindle density was calculated as the ratio of the number of sleep spindles counted in N2-sleep to the number of minutes of N2-sleep. REM-sleep was classified according to the standard criteria of rapid eye movements, low muscle tone, and rapid low-voltage EEG and was monitored online by two trained psychologists. As soon as the first 30-s REM-sleep epoch was identified, participants were awakened. At the beginning of the night, it was usually sufficient to awaken the participants using an intercom. If participants did not wake up completely this way, one of the investigators went to the participant's room and addressed him or her personally. If this was not sufficient either, the participants were asked to sit up for a moment before being allowed to sleep again.

### Statistical Analysis

The manipulation check of the REM-sleep deprivation was performed using two-sample t-tests. A mixed ANOVA model with the between subjects factor GROUP (control versus REMD) and the within subjects factors CONDITION (wake versus sleep) and AFFECT (emotional versus neutral) was used to assess the effect of REM-deprivation and sleep on the consolidation of the (emotional) stimulus material. The degrees of freedom for within-subject effects were corrected according to Greenhouse-Geisser. T-tests were carried out as *post-hoc* tests. The level of significance was set at 5%. Data analysis was performed with SPSS for Windows, version 20.0 (SPSS Inc., Chicago, IL, USA).

## Results

### Manipulation Check

**Figure 1:**
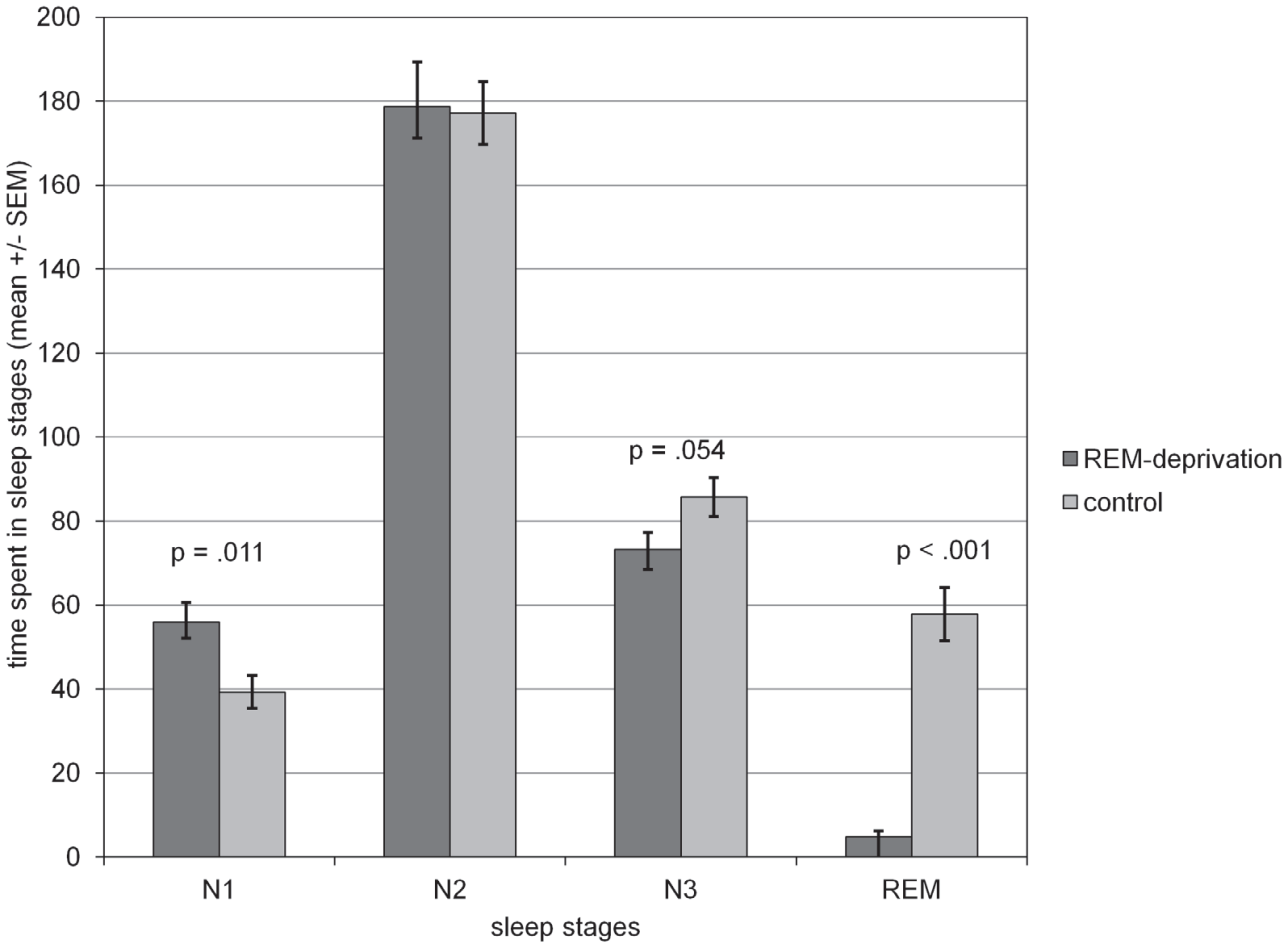
Manipulation check. Minutes in sleep stages N1, N2, N3 and REM sleep (mean ±SEM). Participants in the REM-sleep deprivation condition remained in REM sleep for significantly less time and more in N1 than those in the control group. (* p<.05; ** p<.001).

When comparing the percentage of REM sleep across the two nights for each experimental group we found a highly significant decrease of REM sleep from 11.9%±1.2 (mean ±SEM; adaptation night) to 1.4%±.4 (experimental night) in the REM deprivation group (p<.001; paired t-test) and a slight increase in the undisturbed sleep group (12.4%±1.2 and 15.7%±1.4; p = .07). Values of sleep parameters are shown in Figure 1 and Table 3. The deprivation group had only 4.8±1.5 min (mean ±SEM) of REM-sleep compared to 57.79±6.34 min in the control group (p<.001). Both groups had nearly the same amount of N2-sleep. The REM-sleep deprived group had 12.5 min less N3-sleep, which is 14.6% less than in the control group [t(27) = 2.02, p = .054]. Participants of the REM-deprivation group showed more light sleep (N1) than the control group. Furthermore, there was no significant difference in total non-REM-sleep between the groups [t(27) = −0.44, p = .664].

**Table 3:** Group comparisons between REM-deprivation group and control group with respect to sleep parameters.

	REM deprivation group Mean (SEM)	Undisturbed sleep group Mean (SEM)	t	df	p
Total sleep time (min)	311 (11)	360 (11)	3.15	27	0.004
Wake after sleep onset (min)	93 (7)	40 (7)	−5.17	27	<0.001
N1 (min)	56 (5)	39 (4)	−2.72	27	0.01
N2 (min)	179 11)	177 (7)	−0.11	25	0.91
N3 (min)	73 (4)	86 (5)	2.02	27	0.054
REM sleep (min)	5 (2)	58 (6)	8.13	14	<0.001
Sleep spindle density (%)	4.8 (0.4)	4.1 (0.4)	−1.21	27	0.24

Sleep spindle density in % (per minute of stage 2 sleep; C4-A1).

The manipulation of emotional arousal induced by neutral versus emotional pictures was also successful. In Picture Set 1, the rated arousal differed significantly between emotional and neutral pictures [t(12) = 9.55, p<.001]. In Picture Set 2, the ratings for emotional and neutral pictures also differed significantly [t(15) = 8.03, p<.001]. The observed significant differences in ratings for emotional and neutral pictures also existed for comparison (mean ±SEM) in the wake (emotional: 5.71±0.31, neutral: mean = 2.59±0.21) and sleep (emotional: mean = 5.64±0.31, neutral: mean = 2.66±0.22) conditions.

Before the learning phase in both wake and sleep conditions, participants took part in the Digit Span memory task. The total score of this task did not differ significantly between the experimental groups in the wake and sleep condition, showing similar attention and short-term memory in both groups (p>.21). Our data also showed no significant differences between both groups in the arousal and valence ratings in the morning after the experimental night (Table 2).

### Effects of Sleep on Memory

**Figure 2:**
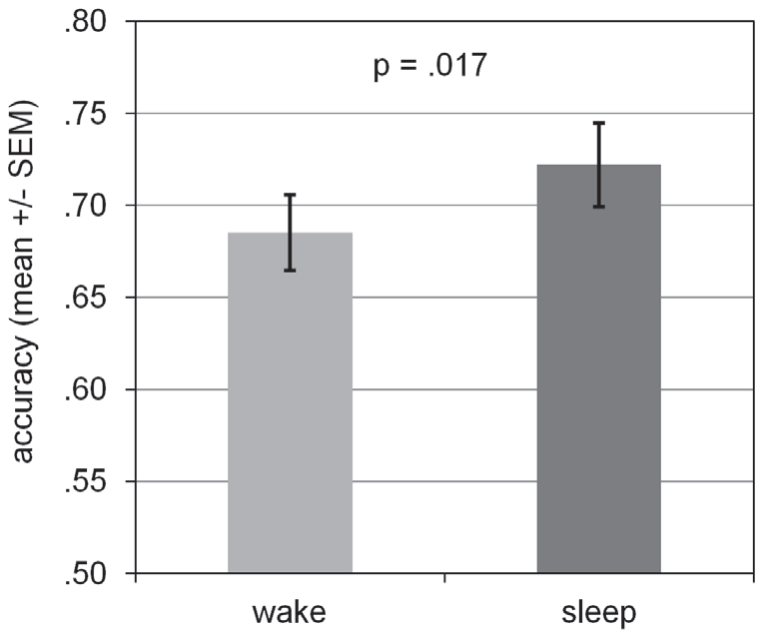
Accuracy for pictures (emotional and neutral taken together) in both conditions (wake vs. sleep). Participants showed a significantly higher accuracy in the condition sleep relative to wake (*p<.05).

**Figure 3:**
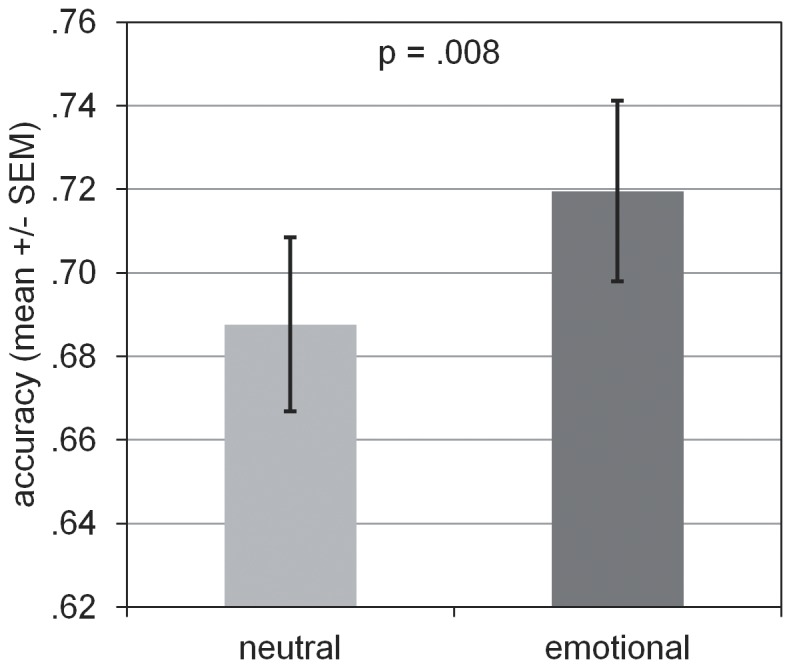
Accuracy for emotional and neutral pictures at retrieval (sleep and wake condition taken together). Participants showed a significant higher accuracy for emotional versus neutral pictures (*p<.05).

The main question was whether (REM-) sleep deprivation diminishes the consolidation of (emotional) pictures which should result in lower accuracy scores in the recognition test after the retention interval. An ANOVA revealed a significant main effect for the factor SLEEP (wake vs. sleep) [F(1,27) = 6.52, p = .017]. Figure 2 shows that recognition accuracy for pictures (emotional + neutral) was higher after a night of sleep than after a retention interval spent awake. The expected main effect for the factor AFFECT (emotional vs. neutral) also was significant [F(1,27) = 8.21, p = .008]. Memory for emotional pictures was better than for neutral ones (Fig. 3). There was no significant main effect for the factor DEPRIVATION (control vs. REMD) [F(1,27) = 0.003, p = .816]. Furthermore, all interaction effects were not significant [SLEEP * DEPRIVATION: F(1,27) = 0.047, p = .831; SLEEP * AFFECT: F(1,27) = 0.969, p = .334; DEPRIVATION * AFFECT: F(1,28) = 3.295, p = .081; SLEEP * AFFECT * DEPRIVATON: F(1,27) = 0.387, p = .539]. That is, we did not find a selective effect of REM-sleep deprivation on the consolidation of emotional content. Values of accuracy and post-hoc t-tests are shown in Table 4. None of the single comparisons was significant (p>.16). Correlations between recognition accuracy (neutral and emotional memory) and sleep stage N3 or sleep spindle density were also not significant (p>.1).

**Table 4:** Comparison of accuracy between both experimental groups.

		REM-deprivation group	Undisturbed sleep group			
		M(SEM)	M(SEM)	t	df	p
**wake condition**	neutral pictures	.641 (.030)	.683 (.030)	0.982	27	.168
	emotional pictures	.716 (.032)	.700 (.031)	−0.366	27	.359
**sleep condition**	neutral pictures	.704 (.029)	.722 (.041)	0.362	27	.360
	emotional pictures	.733 (.035)	.729 (.037)	−0.093	27	.464

Accuracy =  difference between hit rate and false-alarm rate.

## Discussion

The aim of this study was to specify the role of REM-sleep in the consolidation of emotional memory. Using the technique of selective REM-sleep deprivation, we investigated whether REM-sleep is necessary to consolidate declarative memories and, more specifically, whether REM-sleep is necessary to enhance consolidation of emotional compared to neutral content.

We replicated the effect that sleep fosters the consolidation of declarative memories [23], regardless of the emotional arousal attached to the stimuli. After an 8 h retention interval of a night of sleep, our participants performed better in the recognition test than after a wake retention interval of the same length. Furthermore, we replicated the memory-enhancing effect of emotional arousal [24]. In both conditions (wake and sleep), emotional pictures were better recognized than neutral pictures.

Emotional memory in particular is supposed to benefit from sleep after encoding [3]–[4], [10], [25]. Wagner *et al.* [3] used the Ekstrand paradigm (early vs. late night sleep) and found that sleep selectively favors the retention of emotional texts relative to neutral texts and that this benefit was only present following late-night-sleep (rich of REM-sleep). In their nap-paradigm Nishida *et al.* [8] used negative emotional and neutral pictures and found a selective offline emotional memory advantage correlated with the amount of REM-sleep and the extent of right-dominant prefrontal theta waves during this sleep stage.

In two other studies, a benefit of sleep (especially for emotional memory) was found only for high confidence answers but not for recognition accuracy in total. Hu *et al*. [10] failed to find a preferential benefit of the sleep condition on accuracy for arousal stimuli relative to neutral stimuli for “remember” judgments (low confidence answers). They only found this benefit for “know” judgments (high confidence answers). Also, Groch *et al*. [7] showed a significant difference only for answers with high confidence. For all answers (including low-confidence responses), the observed advantage of memory for emotional stimuli over neutral ones was only significant for the hit-rate results but not for accuracy (hit-rate – false-alarm rate).

Our findings are coherent with some recent research which also failed to show a selective emotional memory enhancing effect of (REM-) sleep. Lewis *et al*. [6] showed that context memory decayed less across an overnight retention interval containing sleep relative to an equivalent retention interval containing daytime wakefulness. They found that emotional content of contextual memories did not interact with the reduction in forgetting. Baran *et al.* [11] used a memory paradigm similar to ours in which their participants learned negative and neutral IAPS-pictures [12]. When comparing a wake and sleep condition, they also found better recognition memory following sleep compared to wake only for emotional and neutral pictures together however, their data did not show a relationship of memory and measures of REM either.

Different attempts have been made to analyze the functional significance of neuronal and hormonal processes known to take place during REM-sleep by pharmacological or behavioral suppression of REM-sleep [26]. Studies with selective REM-sleep deprivation provided indications of a role of REM sleep concerning consolidation of procedural memory in humans [27]–[28]. The technique of REM-sleep deprivation is therefore an interesting approach to also analyze sleep-related emotional memory processes. In this study, we successfully employed a rigorous technique of selective REM-sleep deprivation in which the amount of REM-sleep during an eight hour sleep window was minimized to a mean of 4.8 min. Furthermore, the procedure did not affect the total amount of non-REM-sleep. Compared to the technique of partial REM-sleep deprivation using the split-night paradigm [3], [29], we obtained a much better reduction of REM-sleep. Therefore, any proposed effect of REM-sleep on consolidation should be much stronger than in studies using the split-night paradigm or studies which simply correlate the relative amount of REM-sleep with performance. Moreover, we used comparable or even larger sample sizes and a comparable number of items in the memory test as other studies in the field [6]–[8]. Despite the fact that our study should have more power than previous studies, we did not find the expected effect of REM-sleep deprivation on memory consolidation.

Nevertheless, several authors argued that the technique of selective REM-sleep deprivation and the repeated disturbance of sleep might cause stress and therefore diminish memory retrieval [7], [30]. The participants' sensation of stress accompanied with the repeated awakenings could lead to an increase in cortisol levels. There are contradicting results concerning the influence of cortisol on memory [31]. Increasing glucocorticoid levels during sleep by post-learning administration of dexamethasone impaired consolidation of declarative memory [32]. Cortisol suppression with metyrapone also impaired retention of neutral stimuli without altering the recognition of emotional memory [33]. In a study by Gonnissen *et al.* [34], sleep fragmentation due to awakenings every 90 minutes during the night caused a significant but slight reduction of waking cortisol in the morning. More pronounced fragmentation with arousals every 30 seconds lead to an increase in morning cortisol measured after the second night [35]. Decreased plasma cortisol levels have been reported in a former study with REM-sleep deprivation [36]. The self-evaluated emotional and arousal state of participants, which could be influenced by the stress due to awakenings in the REM-deprived group, did not differ significantly between both experimental groups as measured by self-reporting of valence and arousal and therefore most likely did not influence memory consolidation.

Our study may be limited by the use of only negative emotions. Indeed, this is a common method to manipulate emotional arousal but limits the results to this state of emotion. Another limitation is that our encoding control only consisted of 20 pictures and was therefore too low for an acceptable baseline measure. A further limitation is that our selective REM-sleep paradigm was successful at reducing REM-sleep, but also produced a significant increase in light sleep (N1) and a significant decrease in total sleep time and in SWS. For a more detailed analysis of possible memory effects of SWS, an SWS-deprived control group would be helpful and is a promising research step in the future, and results of a study by Groch *et al.* [37] also point in this direction.

Another explanation of our results is that other processes that are important to memory consolidation and normally associated with REM-sleep, such as high cholinergic activity or coherent theta activity in amygdala and PFC [1], [24], [38], may persist during REM-sleep deprivation and thereby result in consolidation of emotional memory during sleep. It is also possible that other aspects of sleep, which are undisturbed by REM-sleep deprivation, such as stage 2 sleep spindles, may also be important in the processing of emotional memory. A possible role of sleep spindles for emotional memory performance, however, was not supported by our own correlational analyses but by a recent pharmacology study [39]. In this study hypnotics increased sleep spindle density and enhanced recognition of negative and high-arousal memories. These results raise the possibility that sleep spindles may causally facilitate emotional memory consolidation. However, further studies are required to elucidate possible interactions between sleep spindles and emotional memory processes (e.g. pharmacological enhancement of spindle activity in REM-sleep deprivation).

In summary, our data suggest, that REM-sleep deprivation was successful and that the resulting massive reduction in REM-sleep had no influence on memory consolidation whatsoever. It seems that sleep-dependent emotional memory consolidation does not solely rely on intact amounts of REM-sleep throughout a night of sleep.

## Supporting Information

Supplements S1 **File containing Tables S1–S4.** Table S1a: Accuracy. ANOVA with the within-subject factors SLEEP (sleep vs. wake), AFFEKT (negative vs. neutral) and the between-subject factor DEPRIVATION (REMS-deprived vs. undisturbed). Table S1b: Accuracy. Descriptives. Table S2a Hit rates. ANOVA with the within-subject factors SLEEP (sleep vs. wake), AFFEKT (negative vs. neutral) and the between-subject factor DEPRIVATION (REMS-deprived vs. undisturbed). Table S2b Hit rates. Descriptives. Table S3a Sensitivity d′. ANOVA with the within-subject factors SLEEP (sleep vs. wake), AFFEKT (negative vs. neutral) and the between-subject factor DEPRIVATION (REMS-deprived vs. undisturbed). Table S3b Sensitivity d′. Descriptives. Table S4a Response bias c. ANOVA with the within-subject factors SLEEP (sleep vs. wake), AFFEKT (negative vs. neutral) and the between-subject factor DEPRIVATION (REMS-deprived vs. undisturbed). Table S4b Response bias c. Descriptives.
(DOCX)
