# Supplementary material for: Selective REM-Sleep Deprivation Does Not Diminish Emotional Memory Consolidation in Young Healthy Subjects
Source: PLoS One. 2014 Feb 27;9(2):e89849. doi: 10.1371/journal.pone.0089849 (PMC3937423; doi:10.1371/journal.pone.0089849)
Supplement: Supplements S1 — File containing Tables S1–S4. Table S1a: Accuracy. ANOVA with the within-subject factors SLEEP (sleep vs. wake), AFFEKT (negative vs. neutral) and the between-subject factor DEPRIVATION (REMS-deprived vs. undisturbed). Table S1b: Accuracy. Descriptives. Table S2a Hit rates. ANOVA with the within-subject factors SLEEP (sleep vs. wake), AFFEKT (negative vs. neutral) and the between-subject factor DEPRIVATION (REMS-deprived vs. undisturbed). Table S2b Hit rates. Descriptives. Table S3a Sensitivity d′. ANOVA with the within-subject factors SLEEP (sleep vs. wake), AFFEKT (negative vs. neutral) and the between-subject factor DEPRIVATION (REMS-deprived vs. undisturbed). Table S3b Sensitivity d′. Descriptives. Table S4a Response bias c. ANOVA with the within-subject factors SLEEP (sleep vs. wake), AFFEKT (negative vs. neutral) and the between-subject factor DEPRIVATION (REMS-deprived vs. undisturbed). Table S4b Response bias c. Descriptives. (DOCX) [file pone.0089849.s001.docx]

Table S1a: Accuracy. ANOVA with the within-subject factors SLEEP (sleep vs. wake), AFFEKT (negative vs. neutral) and the between-subject factor DEPRIVATION (REMS-deprived vs. undisturbed)

| **Source** | **SS** | **df** | **MS** | **F** | **p** |
| --- | --- | --- | --- | --- | --- |
| SLEEP | 0.039 | 1 | 0.039 | 6.520 | .017 |
| SLEEP * DEPRIVATION | < 0.001 | 1 | < 0.001 | 0.047 | .831 |
| Error | 0.163 | 27 | 0.006 |  |  |
| AFFEKT | 0.029 | 1 | 0.029 | 8.206 | .008 |
| AFFEKT * DEPRIVATION | 0.012 | 1 | 0.012 | 3.295 | .081 |
| Error | 0.097 | 27 | 0.004 |  |  |
| SLEEP * AFFEKT | 0.006 | 1 | 0.006 | 0.969 | .334 |
| SLEEP * AFFEKT * DEPRIVATION | 0.002 | 1 | 0.002 | 0.387 | .539 |
| Error | 0.161 | 27 | 0.006 |  |  |
| DEPRIVATION | 0.003 | 1 | 0.003 | 0.055 | .816 |
| Error | 1.314 | 27 | 0.049 |  |  |

Table S1b: Accuracy. Descriptives

| **DEPRIVATION** | **SLEEP** | **AFFEKT** | **Mean** | **SEM** |
| --- | --- | --- | --- | --- |
| undisturbed | wake | neutral | 0.683 | 0.030 |
|  |  | negative | 0.700 | 0.031 |
|  | sleep | neutral | 0.722 | 0.041 |
|  |  | negative | 0.729 | 0.037 |
| REMS-deprived | wake | neutral | 0.641 | 0.030 |
|  |  | negative | 0.716 | 0.032 |
|  | sleep | neutral | 0.704 | 0.029 |
|  |  | negative | 0.733 | 0.035 |

Table S2a: Hit rates. ANOVA with the within-subject factors SLEEP (sleep vs. wake), AFFEKT (negative vs. neutral) and the between-subject factor DEPRIVATION (REMS-deprived vs. undisturbed)

| **Source** | **SS** | **df** | **MS** | **F** | **p** |
| --- | --- | --- | --- | --- | --- |
| SLEEP | 0.019 | 1 | 0.019 | 2.596 | .119 |
| SLEEP * DEPRIVATION | 0.004 | 1 | 0.004 | 0.508 | .482 |
| Error | 0.198 | 27 | 0.007 |  |  |
| AFFEKT | 0.033 | 1 | 0.033 | 9.512 | .005 |
| AFFEKT * DEPRIVATION | 0.003 | 1 | 0.003 | 0.906 | 0.350 |
| Error | 0.094 | 27 | 0.003 |  |  |
| SLEEP * AFFEKT | 0.003 | 1 | 0.003 | 1.459 | .238 |
| SLEEP * AFFEKT * DEPRIVATION | 0.002 | 1 | 0.002 | 0.804 | .378 |
| Error | 0.05 | 27 | 0.002 |  |  |
| DEPRIVATION | 0.010 | 1 | 0.010 | 0.249 | .622 |
| Error | 1.049 | 27 | 0.039 |  |  |

Table S2b: Hit rates. Descriptives

| **DEPRIVATION** | **SLEEP** | **AFFEKT** | **Mean** | **SEM** |
| --- | --- | --- | --- | --- |
| undisturbed | wake | neutral | 0.765 | 0.029 |
|  |  | negative | 0.791 | 0.027 |
|  | sleep | neutral | 0.782 | 0.033 |
|  |  | negative | 0.803 | 0.032 |
| REMS-deprived | wake | neutral | 0.754 | 0.028 |
|  |  | negative | 0.816 | 0.026 |
|  | sleep | neutral | 0.808 | 0.032 |
|  |  | negative | 0.835 | 0.031 |

Table S3a: Sensitivity d‘. ANOVA with the within-subject factors SLEEP (sleep vs. wake), AFFEKT (negative vs. neutral) and the between-subject factor DEPRIVATION (REMS-deprived vs. undisturbed)

| **Source** | **SS** | **df** | **MS** | **F** | **p** |
| --- | --- | --- | --- | --- | --- |
| SLEEP | 1.416 | 1 | 1.416 | 10.259 | .003 |
| SLEEP * DEPRIVATION | 0.047 | 1 | 0.047 | 0.337 | .566 |
| Error | 3.726 | 27 | 0.138 |  |  |
| AFFEKT | 0.433 | 1 | 0.433 | 3.367 | .078 |
| AFFEKT * DEPRIVATION | 0.331 | 1 | 0.331 | 2.579 | .120 |
| Error | 3.470 | 27 | 0.129 |  |  |
| SLEEP * AFFEKT | 0.163 | 1 | 0.163 | 0.983 | .330 |
| SLEEP * AFFEKT * DEPRIVATION | 0.068 | 1 | 0.068 | 0.406 | .529 |
| Error | 4.490 | 27 | 0.166 |  |  |
| DEPRIVATION | 0.555 | 1 | 0.555 | 0.685 | .415 |
| Error | 21.883 | 27 | 0.810 |  |  |

Table S3b: Sensitivity d‘. Descriptives

| **DEPRIVATION** | **SLEEP** | **AFFEKT** | **Mean** | **SEM** |
| --- | --- | --- | --- | --- |
| undisturbed | wake | neutral | 2.252 | 0.129 |
|  |  | negative | 2.294 | 0.140 |
|  | sleep | neutral | 2.540 | 0.156 |
|  |  | negative | 2.528 | 0.167 |
| REMS-deprived | wake | neutral | 1.998 | 0.125 |
|  |  | negative | 2.351 | 0.136 |
|  | sleep | neutral | 2.303 | 0.151 |
|  |  | negative | 2.408 | 0.162 |

Table S4a: Response bias c. ANOVA with the within-subject factors SLEEP (sleep vs. wake), AFFEKT (negative vs. neutral) and the between-subject factor DEPRIVATION (REMS-deprived vs. undisturbed)

| **Source** | **SS** | **df** | **MS** | **F** | **p** |
| --- | --- | --- | --- | --- | --- |
| SLEEP | 0.007 | 1 | 0.007 | 0.108 | .745 |
| SLEEP * DEPRIVATION | 0.078 | 1 | 0.078 | 1.288 | .266 |
| Error | 1.633 | 27 | 0.060 |  |  |
| AFFEKT | 0.116 | 1 | 0.116 | 1.952 | .174 |
| AFFEKT * DEPRIVATION | 0.003 | 1 | 0.003 | 0.045 | .833 |
| Error | 1.600 | 27 | 0.059 |  |  |
| SLEEP * AFFEKT | < 0.001 | 1 | < 0.001 | < 0.001 | .992 |
| SLEEP * AFFEKT * DEPRIVATION | 0.004 | 1 | 0.004 | 0.121 | .731 |
| Error | 0.914 | 27 | 0.034 |  |  |
| DEPRIVATION | 0.404 | 1 | 0.404 | 1.792 | .192 |
| Error | 6.086 | 27 | 0.225 |  |  |

Table S4b: Response bias c. Descriptives

| **DEPRIVATION** | **SLEEP** | **AFFEKT** | **Mean** | **SEM** |
| --- | --- | --- | --- | --- |
| undisturbed | wake | neutral | -0.363 | 0.072 |
|  |  | negative | -0.277 | 0.079 |
|  | sleep | neutral | -0.387 | 0.092 |
|  |  | negative | -0.327 | 0.084 |
| REMS-deprived | wake | neutral | -0.275 | 0.070 |
|  |  | negative | -0.233 | 0.077 |
|  | sleep | neutral | -0.219 | 0.089 |
|  |  | negative | -0.154 | 0.081 |
